# Modeling to inform strategies for malaria eradication

**DOI:** 10.1186/1475-2875-13-S1-O15

**Published:** 2014-09-22

**Authors:** Steven E Kern

**Affiliations:** 1 Bill & Melinda Gates Foundation, Seattle, Washington USA & University of Utah, Salt Lake City, Utah USA

## 

Mathematical modeling can serve to inform strategies for malaria eradication from different levels of scale depending on the needs of the strategy and the granularity of the data available to create the models. At the most granular level of data, estimates of individual dose-effect relationships for anti-malarial drugs and vaccines can be used to simulate the impact of different implementation strategies for administration and estimate the resulting impact of the therapeutic on disease treatment and transmission reduction. Coupled with epidemiological models that simulate the overall impact on disease prevalence across broad geometries, these tools can become important in planning at a regional level the appropriate response to eradicating malaria. Simplifying assumptions that make the modeling tractable can also limit its usefulness however, as understanding not only the human transmission aspect of the disease but also the vector transfer, the role of underlying geography and weather on vector movement, and the interactions of humans across space and time is important for realistic estimates of strategy impact through modeling. An iterative approach is needed where models predict the impact of an approach, the approach is attempted, the results are fed back to update the model in an iterative way, and new estimates are produced. Through this effort, models of malaria eradication can be important tools in guiding disease eradication.

